# Parkinson's Disease With Visual Hallucinations Is Associated With Epileptiform Activity on EEG

**DOI:** 10.3389/fneur.2021.788632

**Published:** 2022-01-11

**Authors:** Adam Fry, Dharampreet Singh, Louis Manganas, Marc L. Gordon, Christopher Christodoulou, Hoi-Chung Leung, Guy J. Schwartz

**Affiliations:** ^1^Department of Rehabilitation and Human Performance, Icahn School of Medicine at Mount Sinai, New York, NY, United States; ^2^Department of Neurology, Renaissance School of Medicine at Stony Brook University, New York, NY, United States; ^3^Department of Neurology, Joan C. Edwards School of Medicine at Marshall University, Huntington, WV, United States; ^4^The Litwin-Zucker Research Center, The Feinstein Institutes for Medical Research, Northwell Health, New York, NY, United States; ^5^Departments of Neurology and Psychiatry, Donald and Barbara Zucker School of Medicine at Hofstra/Northwell, New York, NY, United States; ^6^Department of Neuropsychology, Renaissance School of Medicine at Stony Brook University, New York, NY, United States; ^7^Department of Psychology, Stony Brook University, Stony Brook, NY, United States

**Keywords:** electroencephalography, non-motor, Parkinson's disease, psychosis, visual hallucinations

## Abstract

**Background:** Visual hallucinations (VHs) in Parkinson's disease (PD) are the cardinal symptoms which declare the onset of PD psychosis (PDP). The anthropomorphic and zoomorphic VHs of PD resemble those of Charles Bonnet syndrome and temporal lobe epilepsy. In both of these disorders electroencephalography (EEG) abnormalities have been described. We therefore sought to examine whether VHs in PD were associated with similar EEG abnormalities.

**Methods:** This retrospective observational study searched the medical records of 300 PD patients and filtered for those containing clinical 20-min scalp EEGs. Remaining records were separated into two groups: patients with reported VHs and those without. The prevalence of epileptiform discharges in the EEGs of both groups was identified.

**Results:** Epileptiform discharges were present in 5 of 13 (38.5%) PD patients with VHs; all localized to the temporal lobe. No epileptiform discharges were observed in the EEGs of the 31 PD patients without VHs.

**Conclusion:** The significantly high incidence of temporal lobe epileptiform discharges in PD patients with VHs as compared to those without VHs lends to the possibility of an association visual cortex epileptogenic focus. Accordingly, for treatment-refractory patients, antiepileptic drugs might be considered, as in the case of Charles Bonnet syndrome, temporal lobe epilepsy and migraine with visual aura. Future prospective studies involving larger samples and multi-center cohorts are required to validate these observational findings.

## Introduction

The psychosis of Parkinson's disease (PD) is one of the most challenging non-motor comorbidities to treat. Complex visual hallucinations (VHs) of people and animals, as well as multimodal hallucinations and delusions are characteristic of Parkinson's disease psychosis (PDP). These VHs are the most common phenomenon in PDP with a lifetime prevalence of nearly 50% (1). As a symptom of psychosis, VHs are the single most reliable predictor of future nursing home institutionalization (2). Despite their prevalence and impact on prognosis, the biologic underpinning of VHs remains uncertain.

Since the time of Hughlings Jackson in the 19th century it became widely accepted that complex VHs originate in the temporal lobe (3). In the 1960s Penfield and Perot demonstrated that seizures and *in vivo* cortical stimulation of this region give rise to complex VHs (4). In more recent years magnetic resonance imaging has demonstrated gray matter volume loss in visual regions, such as the lingual gyrus, in non-demented PD patients with VHs (5). Single photon emission computed tomography has demonstrated perfusion changes within visual association regions in PD patients with VHs (6). Post-mortem histopathological findings show that VHs in PD correlate with temporal lobe cortical Lewy body disease burden (7). Thus, there is a converging lineage of scientific evidence to suggest that the visual association region, which is central in visual integration, may very likely be the pathoanatomical substrate of VHs in PD.

The phenomenology of VHs in PD resembles that of the Charles Bonnet syndrome, a condition causing VHs in the blind. In both conditions the VHs are a paroxysmal phenomenon of well-formed anthropomorphic and zoomorphic images, lasting seconds to minutes in duration (8). As such, they also resemble the visual auras and ictal hallucinations of temporal lobe epilepsy (9). In addition to temporal lobe epilepsy, epileptiform activity has been reported in other disorders which manifest complex visual hallucinations including *secondary causes* of Charles Bonnet syndrome (e.g., from cerebrovascular disease, seizure, etc.) and the Heidenhain variant of Creutzfeldt-Jakob disease (10, 11). Based on these observations, we conducted this retrospective study to examine our hypothesis that epileptiform activity would be present in a subset of patients with PD who report VHs.

## Materials and Methods

This retrospective observational study was reviewed and approved by the Stony Brook University Hospital IRB. The first 300 patients from the Stony Brook Parkinson's and Movement Disorders Center who met the U.K. Parkinson's Disease Society Brain Bank Diagnostic Criteria (12), and who were evaluated at least once by the corresponding author (GS), were selected for inclusion in this study. Through chart review we identified patients with PD who had undergone at least one clinical 20-min scalp EEG. In all cases the EEG was performed to determine whether an epileptic syndrome was the cause of unexplained loss of consciousness, impaired awareness, altered mental status, or abnormal movements. All EEG recordings were performed using the International 10–20 System. Intermittent photic stimulation and hyperventilation were inconsistently performed. The technician who performed the EEG did not document and patients were not interviewed posttest as to whether they experienced VHs during the test. The raw data were interpreted by board-certified epileptologists who received minimal written information about the indication for the test, as is customary in our institution. Their reports were reviewed retrospectively for the purpose of this study. Epileptiform discharges were defined as periodic discharges, rhythmic delta activity, spike-and-wave and sharp-and-wave according to the American Clinical Neurophysiology Society's standardized terminology (13).

Those with a preexisting diagnosis of epilepsy, seizure disorder or another cause that can result in EEG abnormalities were excluded. Those taking at least one antiepileptic drug were also excluded to avoid confounding EEG changes. Included were only those patients with PD whose symptoms-onset appeared before the date of the EEG. Since the yield of detecting abnormal findings increases with serial EEG, for the purpose of uniformity only the first EEG was included in the analysis when multiple EEG were performed for a given patient. We designated the date of the EEG as the end date of the analysis. Age, disease duration, treatment duration and other continuous variables were calculated in relation to this date. Information as to whether a decision was subsequently made to treat with an antiepileptic drug was not captured in our data.

VHs were defined as self- or caregiver-reported zoomorphic, anthropomorphic, or other similar visual phenomena. Chi-squared tests were used to compare categorical variables between patients with and without VHs including sex, self- or caregiver-reported cognitive impairment, antiparkinsonian medications, and epileptiform discharges on EEG. Here, exact *p*-values were calculated using Monte Carlo simulation. Non-parametric Mann-Whitney U tests was used to compare continuous variables including age, age at onset of PD, age at the time of EEG, disease duration, and treatment duration between patients with and without VHs. Results are reported as the median and interquartile range (IQR). All analyses were performed using SAS 9.4 (SAS Institute Inc., Cary, NC).

## Results

**Figure 1:**
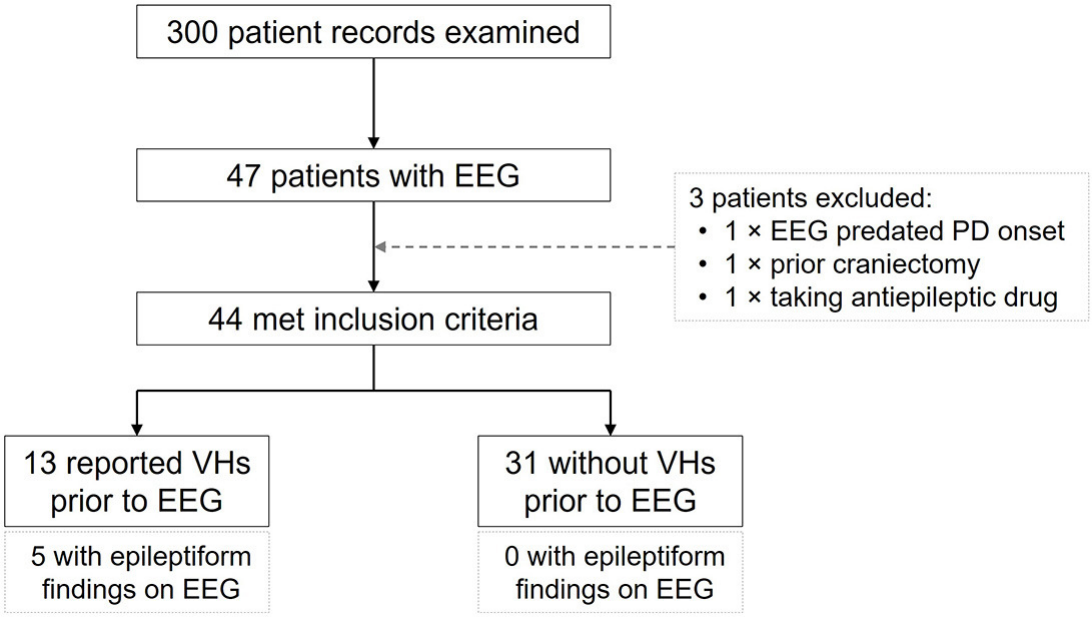
Patient selection/exclusion and incidence of epileptiform activity on EEG.

Of the 300 patient records examined 47 were found to have undergone at least one clinical 20-min scalp EEG. Three of these did not meet study criteria and were excluded (one EEG was performed prior to PD onset; one had undergone a craniectomy for subdural hemorrhage; and one was receiving treatment with an antiepileptic drug). Of the remaining 44 patients, 13 had reported VHs prior to their EEG and 31 had not (Figure 1). None had a preexisting diagnosis of epilepsy or seizure disorder.

Epileptiform activity was detected in 5 of the 13 patients with VHs (38.5%, 95% CI: 16.6–67.26%; Figure 1), and in all cases the activity was localized to the temporal lobes (two left, two right, and one bilaterally; Table 1). In the 31 patients without VHs epileptiform activity was absent. This difference between the two groups was significant (prevalence ratio = 4.875; *p* < 0.001). There were no ictal phenomena detected in any of the EEGs.

**Table 1:** Characteristics of patients with epileptiform discharges on routine EEG.

	**Patient 1**	**Patient 2**	**Patient 3**	**Patient 4**	**Patient 5**
Age (years)	80	79	86	72	70
Sex	M	F	F	F	F
Cognitive impairment	Not endorsed	Not endorsed	Endorsed	Not endorsed	Not endorsed
PD duration (years)	6	13	16	2	1
Medications	Carbidopa-levodopa	Carbidopa-levodopa, entacapone	Carbidopa-levodopa, selegiline, amantadine	Rasagiline	Carbidopa-levodopa
Treatment duration (years)	6	13	16	2	1
Visual hallucinations	Well-formed	Well-formed	Well-formed	Well-formed	Well-formed
EEG	Right temporal sharp waves	Left temporal sharp waves	Left temporal rhythmic delta with sharp waves	Bilateral temporal sharp waves	Right temporal sharp waves

Sex, age at onset of PD, age at the time of EEG, rate of self- or caregiver-reported cognitive impairment, and use of antiparkinsonian medications did not differ between patients with and without reported VHs (Table 2). However, patients reporting VHs had a significantly longer disease duration (11 years, IQR: 10) than those without VHs (6.5 years, IQR: 9.5; *p* = 0.037). Accordingly, duration of treatment with antiparkinsonian medications was also longer in those with VHs (11 years, IQR: 7) as compared to those without (3 years, IQR: 7 years; *p* = 0.007).

**Table 2:** Patient characteristics and medication use.

	**VH-negative**	**VH-positive**	***p*-value**
**Patient characteristics**			
Females, *n (%)*	12 (38.7)	6 (46.2)	0.647
Age at onset of PD, *median years (IQR)*	68 (12)	66 (12)	0.315
Age at time of antiparkinsonian drugs treatment commencement, *years*	70 (14)	67 (11)	0.176
Age at time of EEG, *median years (IQR)*	73 (6)	75 (9)	0.262
Disease duration at time of EEG, *median years (IQR)*	6 (8)	11 (10)	0.037
Antiparkinsonian drugs treatment duration at time of EEG, *median years (IQR)*	3 (7)	11 (7)	0.007
Cognitive impairment, *n (%)*	7 (22.3)	3 (23.0)	0.974
**Medication use**			
Levodopa, *n (%)*	21 (67.7)	12 (92.3)	0.086
Dopamine receptor agonists, *n (%)*	4 (12.9)	1 (7.7)	0.619
NMDA receptor antagonist, *n (%)*	1 (3.2)	2 (15.3)	0.144
MAO-B inhibitors, *n (%)*	10 (32.2)	5 (38.5)	0.692
COMT inhibitors, *n (%)*	2 (6.5)	1 (7.7)	0.882

## Discussion

In this retrospective observational study examining the initial 20-min scalp EEG of 44 patients with PD we compared the incidence of epileptiform activity in those with VHs to those without. Our main finding was that epileptiform discharges were found in 5 of the 13 patients with VHs (38.5%), and in all such cases they localized to the temporal lobe. Conversely, no EEG epileptiform discharges were identified in the 31 patients without VHs. When compared to prior reports, these results indicate that the incidence of epileptiform discharges in those with PD and VHs may be similar to the geriatric population with new-onset and mostly focal epilepsy [26.3% (14)] and higher than the geriatric population without epilepsy [4.7% (15)].

In comparison to our patients without VHs, patients with VHs had a longer disease duration and treatment course but were similar in age, sex, cognitive impairment status, and medication use. The longer disease duration associated with VHs is in keeping with the observation that although VHs can occur at any stage of the disease, they typically appear in advanced disease (16). Notwithstanding, three of the five patients with epileptiform discharges on EEG exam had disease and treatment durations of ≤ 6 years (Table 1) suggesting that lengthier disease/treatment durations were not responsible for the association between VHs and epileptiform discharges, however, larger cohort studies will be required to formally assess this. Overall, these findings lead to our observation that in non-epileptic patients with PD, VHs are associated with epileptiform discharges within the temporal lobe.

The biologic underpinnings of VHs in PD have not been fully explicated. Medications, the underlying neurodegenerative changes, a complex interrelation between them, incidental comorbid diseases, and other contributing factors have all been proposed as causative. Much emphasis has been made on antiparkinsonian medications and restricting their use has historically been applied to curtailing VHs. However, the role of dopaminergic medications in the potentiation of VHs in PD has been challenged by several authors (16, 17). It is also noteworthy that VHs in PD were reported in the pre-levodopa era and also occur nowadays in drug-naïve patients (18, 19).

Medications that modulate serotoninergic neurons, namely clozapine and pimavanserin, have demonstrable therapeutic benefits (20, 21). Their efficacy might indicate that a neurotransmitter dysfunction is central to the pathogenesis of VHs. But the current standard of care utilizing these two medications is not without its own challenges and shortcomings: a low retention rate due to poor tolerability, low efficacy, onerous blood test monitoring and other factors restricts their use (22). This behooves us to consider novel treatments options.

In 1884, Hughlings Jackson advanced his opinion that visual hallucinations may arise when “some of the cells of the nervous arrangements of these (visual) centers, by any pathological process, become highly unstable to a degree such as occurs in epilepsy.” (3). If so, could VHs in a subset of patients with PD improve with antiepileptic drugs? There is indeed such a precedence in the literature of Charles Bonnet syndrome, temporal lobe epilepsy and visual aura of migraine (23, 24). Thus, it may be worthwhile examining the utility of antiepileptic drugs for treatment-refractory PDP in future studies.

We acknowledge that our findings are subject to several limitations including a high non-response bias of a subjective sensory phenomenon, and unaccounted inter- and intra-reader variability. The use of a low-sensitivity, short duration routine EEG to examine a brief, paroxysmal sensory phenomenon may have also led to underestimations of epileptiform activity prevalence, although we have no reason to believe this would have been more pronounced in those without VHs. Most importantly, as this study involved a retrospective analysis of a convenience sample, we were unable to test causality and the generalizability of our findings is limited. While our results were encouraging as they supported our a-priori hypothesis, prospective studies involving larger cohorts and longer EEG captures are necessary to validate these preliminary findings.

## Data Availability Statement

The original contributions presented in the study are included in the article/supplementary material, further inquiries can be directed to the corresponding author.

## Ethics Statement

The studies involving human participants were reviewed and approved by Stony Brook University Hospital Institutional Review Board. Written informed consent for participation was not required for this study in accordance with the national legislation and the institutional requirements.

## Author Contributions

GS was responsible for the study conception and design. Data collection and analysis were performed by DS and LM. The manuscript was written by AF and GS with substantial review from MG, CC, and H-CL. All authors contributed to the article and approved the submitted version.

## Conflict of Interest

The authors declare that the research was conducted in the absence of any commercial or financial relationships that could be construed as a potential conflict of interest.

## Publisher's Note

All claims expressed in this article are solely those of the authors and do not necessarily represent those of their affiliated organizations, or those of the publisher, the editors and the reviewers. Any product that may be evaluated in this article, or claim that may be made by its manufacturer, is not guaranteed or endorsed by the publisher.
